# Developing Community Advisory Board guidelines for AIDS vaccine trials in China

**DOI:** 10.1186/1742-4690-9-S2-P129

**Published:** 2012-09-13

**Authors:** JA Kaufman, C Liu, A Menezes

**Affiliations:** 1 Harvard Medical School, Boston, MA, USA; 2 Chinese Academy of Medical Sciences, Beijing, China; 3 Global Health Strategies, Rio de Janiero, Brazil

## Background

Community Advisory Boards (CABS) have played an important role in ensuring community oversight of AIDS vaccine trials and are an accepted part of the ethical conduct of AIDS vaccine trials. China's political system limits community engagement in trial communities and wholesale application of western CAB approaches does not accomplish the intent of a CAB. We adapted the CAB concept to China's political system through a multi year process of research and pilot testing in collaboration with six provincial CDCs conducting AIDS prevention research studies.

## Methods

Using questionnaires developed by IAVI for a global CABS study, we carried out an assessment of existing CABS in six provinces in China. The questionnaire examined membership, process, and governance along with other aspects of CABS operations. The results were used to revise standard CABS guidelines developed by IAVI for use in Africa and India. The revised guidelines were pilot tested in three sites, in Yunnan, Guangxi, and Shanxi. In depth interviews were also conducted with CAB members and local CDCS.

## Results

A revised version of Chinese CABS guidelines was published in October 2011 and publicized at China's AIDS Prevention Conference by China's AIDS Ethical Review Board and IAVI. The CABS guidelines are now being promoted as part of the ethical requirements for AIDS prevention clinical trial research by China's National Center for AIDS Prevention and Control.

## Conclusion

Developing and adapting ethical tools for ensuring community input and oversight of clinical trial research in China is necessary given China's political system. These newly adapted CABS guidelines provide a mechanism to accomplish the intention of a CAB even while different in form and governance from standard CABS elsewhere.

